# Extraenteric Malignant Gastrointestinal Neuroectodermal Tumor of the Neck: A Diagnostic Challenge

**DOI:** 10.3390/ijms26104517

**Published:** 2025-05-09

**Authors:** Manuel Tousidonis, Maria J. Troulis, Carolina Agra, Francisco Alijo, Ana Alvarez-Gonzalez, Carlos Navarro-Cuellar, Saad Khayat, Gonzalo Ruiz-de-Leon, Ana Maria Lopez-Lopez, Jose Ignacio Salmeron, Santiago Ochandiano

**Affiliations:** 1 Department of Oral and Maxillofacial Surgery, Gregorio Marañón University Hospital, Calle Doctor Esquerdo 46, 28006 Madrid, Spainruizdeleong@gmail.com (G.R.-d.-L.);; 2 Gregorio Marañón Research Institute, 28009 Madrid, Spain; 3 Department of Oral and Maxillofacial Surgery, Massachusetts General Hospital, Harvard School of Dental Medicine, 188 Longwood Avenue, Boston, MA 02115, USA; mtroulis@mgb.org; 4 Department of Pathology, Gregorio Marañón University Hospital, Calle Doctor Esquerdo 46, 28006 Madrid, Spain; 5 Department of Radiation Oncology, Gregorio Marañón University Hospital, Calle Doctor Esquerdo 46, 28006 Madrid, Spain

**Keywords:** malignant gastrointestinal neuroectodermal tumor, clear cell sarcoma, soft-tissue neoplasm, neck cancer, *EWSR1::CREB*

## Abstract

Malignant gastrointestinal neuroectodermal tumor (MGNET) and clear cell sarcoma (CCS) of soft tissue represent related, extremely rare, malignant mesenchymal neoplasms. Both entities are genetically characterized by the same molecular alterations, *EWSR1::CREB1* fusions. Malignant gastrointestinal neuroectodermal tumor has significant morphological overlap with CCS, although it tends to lack overt features of melanocytic differentiation. Recently, rare MGNET cases were reported in extragastrointestinal sites. The diagnosis represents a major challenge and significantly impacts therapeutic planning. In this study, we reported the clinicopathologic features of a molecularly confirmed MGNET of the neck and provided a review of the pertinent literature.

## 1. Introduction

Malignant gastrointestinal neuroectodermal tumor (MGNET) [1], previously referred to as clear cell sarcoma (CCS)-like tumor of the gastrointestinal tract [2,3,4], is an exceedingly rare and aggressive mesenchymal neoplasm. Malignant gastrointestinal neuroectodermal tumor (MGNET) and clear cell sarcoma (CCS) are rare soft tissue neoplasms that, despite sharing molecular features such as EWSR1 gene rearrangements (*EWSR1-CREB1* in MGNET and *EWSR1-ATF1* in CCS), represent distinct pathological entities. Initially described in the gastrointestinal (GI) tract, particularly in the small intestine and stomach, this tumor has recently been identified in extraintestinal locations, including the head and neck. Occurrences in the head/neck (H/N) region are exceptionally rare, with less than 100 cases cited in the literature worldwide. The first case of MGNET in the H/N was reported by Alpers and Beckstead [1] as a “malignant neuroendocrine tumor of the jejunum with osteoclast-like giant cells”. The etiopathogenesis of MGNET remains poorly understood due to its rarity [5], though several key mechanisms have been identified. MGNET is believed to arise from neuroectodermal cells, which show early neural differentiation [6]. Central to its development are genetic fusions [7], particularly involving *EWSR1::CREB1*, which drive tumor proliferation. The exact environmental or external factors contributing to MGNET are still unknown, although its aggressive behavior and occurrence in both gastrointestinal and extraintestinal sites, including the head/neck, may suggest multiple pathways of tumorigenesis. According to the World Health Organization (WHO) classification of soft tissue tumors [8], MGNET and CCS are categorized within the group of neoplasms with uncertain differentiation. Despite shared molecular features, including *EWSR1::CREB1* fusions and co-expression of S100 protein and SOX10, MGNET and CCS exhibit distinct morphological and immunohistochemical characteristics [9,10,11,12]. Notably, CCS expresses melanocytic markers such as HMB-45, Melan-A, tyrosinase, and MiTF, which are absent in MGNET [13,14,15,16]. This immunophenotypic divergence, combined with distinct anatomical localization and lack of melanocytic differentiation in MGNET, supports the classification of these tumors as separate entities despite occasional diagnostic challenges. These differences have led to the preference for the nomenclature of “MGNET” over “clear cell sarcoma-like tumor” [17].

The identification of MGNETs in the head/neck region presents a novel and complex challenge in oncopathology, given the unique embryological origin and intricate anatomy of this area (Table 1). These tumors are characterized by their neuroectodermal differentiation, distinctive histopathological features, and aggressive clinical behavior, underscoring the importance of prompt diagnosis and intervention.

Histologically, both CCS and MGNET consist of epithelioid and spindle cells with prominent nucleoli, organized into large nodules separated by thick fibrous septa. Contrary to the name, the clear cell component is minimal, with most cells displaying eosinophilic cytoplasm. Multinucleated giant cells are observed in approximately half of CCS cases, and melanin pigment can occasionally be detected in soft tissue CCS. In contrast, MGNET is distinguished by a pseudopapillary and/or pseudoalveolar growth pattern. In this report, a rare case of extraenteric MGNET (E-MGNET) located at the neck is presented, and the clinical, morphological, and molecular features are discussed.

## 2. Case Report

**Figure 1:**
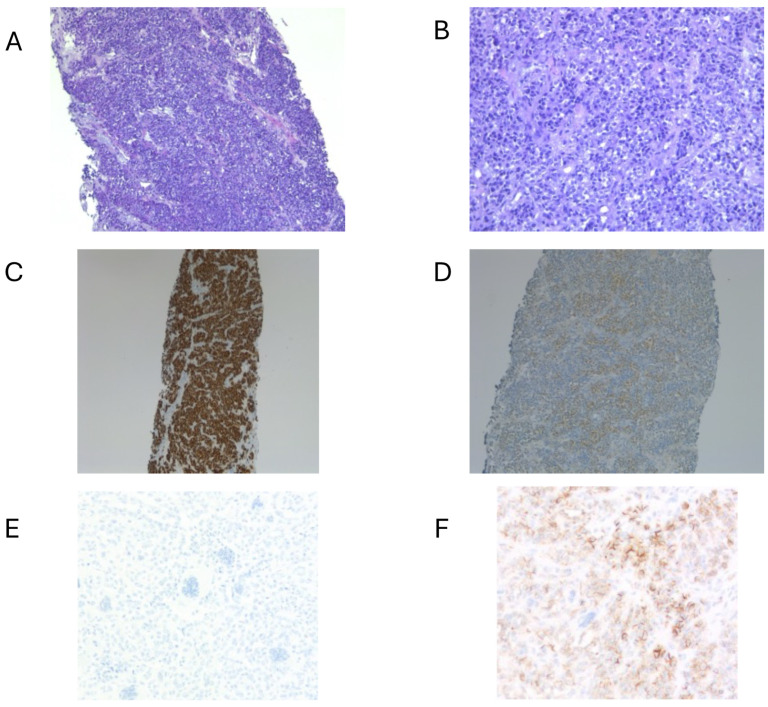
(**A**) Low-power view showing a multinodular, lobulated growth pattern [10×]. (**B**) The tumor is composed of nests and sheets of moderately pleomorphic epithelioid cells with eosinophilic to clear cytoplasm, set in a fibrotic and lymphoplasmacytic stroma [20×]. (**C**,**D**) Immunohistochemically, the tumor cells show diffuse nuclear positivity for SOX10 [10×] (**C**) and cytoplasmic expression of S100 protein [10×] (**D**). (**E**) The neoplastic cells were negative for melanocytic markers, including MiTF, HMB45, and Melan-A [20×]. (**F**) Diffuse membranous expression of CD56 supports neuroectodermal differentiation, consistent with the diagnosis of MGNET [20×].

**Figure 2:**
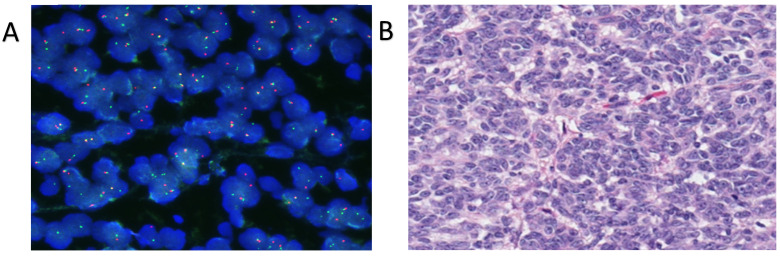
(**A**) At high power, neoplastic cells show vesicular chromatin with small nucleoli (H&E, 400×). (**B**) FISH probe targeting the EWSR1 rearrangement, which revealed red–green split signals in 94% of the tumor cell nuclei, indicating EWSR1 gene rearrangement [20×].

**Figure 3:**
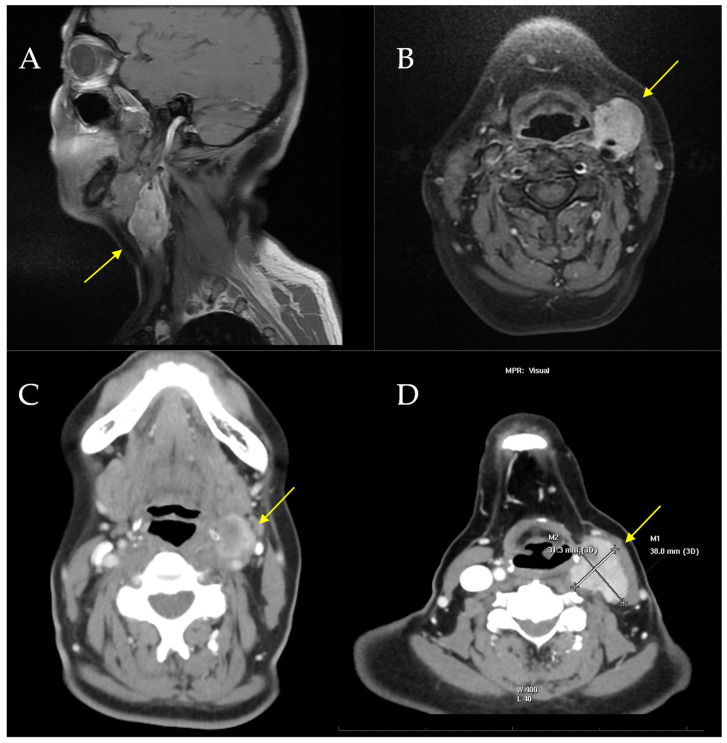
Extraenteric malignant gastrointestinal neuroectodermal tumor involving the soft tissues of the neck in a 58-year-old woman: (**A**) Sagittal MRI image at diagnosis. (**B**) Axial MRI image of the tumor at diagnosis. (**C**) Axial CT image in which vascular invasion by the tumor is observed at diagnosis. (**D**) Tumor measurements in the preoperative CT scan.

**Figure 4:**
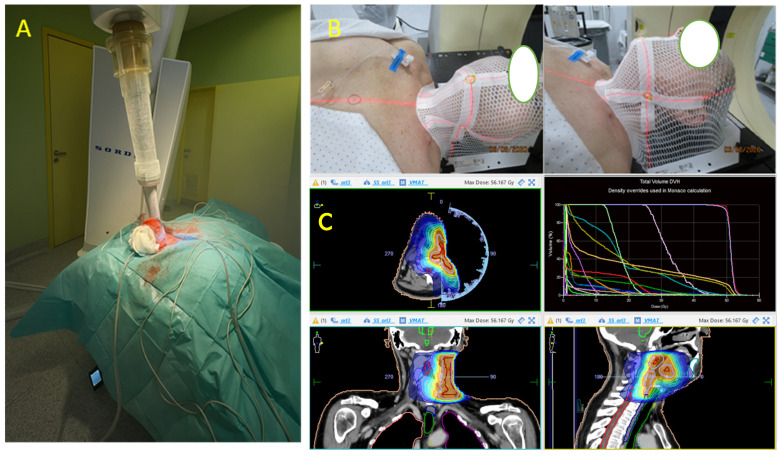
Mixed radiotherapy protocol combining intraoperative radiotherapy (IORT), administered directly to the tumor bed during surgery, followed by postoperative external beam radiotherapy (EBRT) to optimize local disease control. (**A**) Patient setup under general anesthesia for point-of-care photon radiotherapy. (**B**) Placement and fitting of a customized thermoplastic mask on the patient’s face, ensuring stable fixation to the anatomical landmarks. Facial features have been masked for privacy. (**C**) Radiotherapy planning and dose distribution over axial, sagittal, and coronal views, including dose–volume histogram analysis.

A 58-year-old woman, with no relevant medical history, was evaluated at another hospital for a rapidly growing painful left cervical mass, biopsied, and diagnosed with “clear cell sarcoma” in October 2019. No cutaneous lesions were identified, and the patient had no history of malignant melanoma. Subsequently, she was referred for evaluation and a second opinion at the Sarcoma Reference Unit of the Gregorio Marañon University Hospital. A new biopsy was conducted, and histological, immunohistochemical, cytogenetic, and next-generation sequencing studies were performed. The results were that a tumor originating from the left laterocervical region was examined, consisting of two paraffin blocks with nine histological slides (H&E and IHC). Microscopically, the neoplasm infiltrated adjacent soft tissues and lymph nodes, displaying perineural and vascular invasion. The tumor exhibited a solid, pseudoalveolar growth pattern with oval to fusiform cells, eosinophilic cytoplasm, vesicular chromatin, and prominent nucleoli. Mitotic count was assessed in five non-overlapping high-power fields (HPFs) corresponding to a total area of 5 mm^2^ (each HPF ≈ 0.2 mm^2^). An average of 1.4 mitoses/mm^2^ (SD ± 0.5) was identified, with focal tumor necrosis observed. This assessment was performed according to current recommendations for reproducibility in mitotic index evaluation [20,21]. Immunohistochemically, the tumor was positive for S100, SOX10, CD99 (weak/focal), Fli-1, and synaptophysin (focal), but negative for melanocytic and epithelial markers (e.g., Melan-A, HMB-45, CKIT, and AE1/AE3) (Figure 1). The Ki-67 proliferation index was assessed by manual counting in three selected hot spot areas of 1 mm^2^ each, with an average labeling index of 15% (SD ± 3%). Quantification was performed in accordance with current recommendations for reproducible assessment of proliferation indices in diagnostic pathology [20,22]. PAS staining was negative. Fluorescence in situ hybridization (FISH) analysis for *EWSR1* was performed using break-apart probes (*EWSR1* Break Apart Probe, TITAN FISH PROBES, OACP IE LTD, Phoenix House, Monahan Road, T12H1XY, Cork, Ireland) on paraffin-embedded sections (Figure 2A). Fluorescence in situ hybridization (FISH) analysis using a break-apart probe targeting *EWSR1* revealed red–green split signals in 93% of tumor cell nuclei, indicating the presence of an *EWSR1* gene rearrangement (Figure 2B). The *EWSR1* rearrangement was confirmed at the Centro Nacional de Investigaciones Oncologicas (CNIO). However, it was not possible to confirm the identity of the fusion partner. Specifically, RT-PCR analysis using *CREB1*-specific primers did not yield amplification products, suggesting that *CREB1* was not involved as the fusion partner gene in this case. FISH analysis confirmed the presence of an EWSR1 gene translocation, supporting a diagnosis of a malignant gastrointestinal neuroectodermal tumor (MGNET) with neuroectodermal differentiation and no melanocytic differentiation. Imaging showed a single left laterocervical mass that invaded the left common carotid artery with no vascular plane of separation (Figure 3A–D). PET-CT confirmed the absence of distant metastasis, and the decision was made to perform surgical excision. One week before the oncological surgery, a left common carotid occlusion test was performed. The test confirmed that the circle of Willis and the collateral pathways were able to compensate for the loss of blood flow through the affected carotid artery before surgery and that, therefore, ligation of the left common carotid artery infiltrated by the tumor could be performed. Treatment with surgery and intraoperative radiotherapy was planned. Surgery with radical left dissection was performed, including resection of the common carotid artery, internal jugular vein, sternocleidomastoid muscle, spinal nerve, and vagus nerve. After surgical resection, intraoperative radiotherapy was performed on the surgical bed with 10 Gy with 6 MeV electrons (Figure 4A).

**Figure 5:**
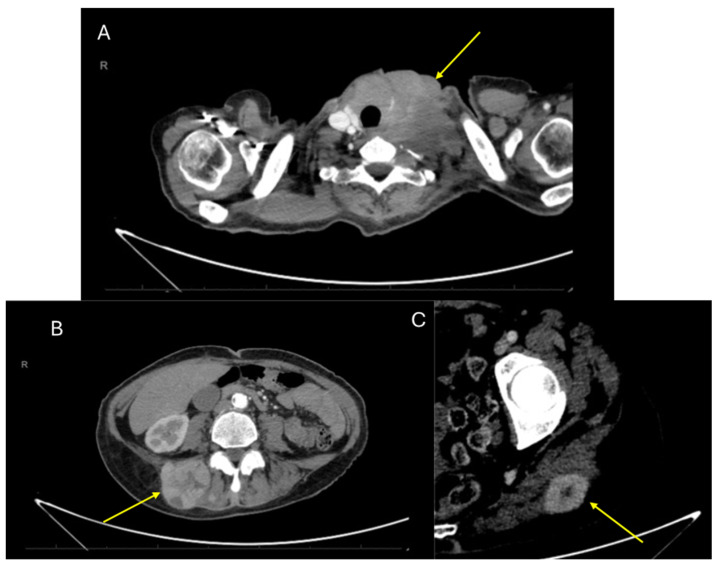
CT images of recurrences during the follow-up period: (**A**) Extensive supraclavicular recurrence 4 years after radical surgery. Absence of the vascular bundle of the neck, resected in radical cervical dissection surgery. (**B**) Contralateral paravertebral metastasis 3 years after diagnosis. (**C**) Ipsilateral gluteal metastasis 3 years after initial diagnosis.

The surgical specimen, “left radical neck dissection”, showed a malignant gastrointestinal neuroectodermal tumor (4 × 2.5 cm), with involvement of the vagus nerve, adventitial layer of the internal carotid artery, and sternocleidomastoid muscle. The surgical resection margin is clear (at >1 mm). Twenty-two lymph nodes measuring between 0.6 and 0.1 cm were isolated, showing nonspecific reactive lymphadenitis, negative for tumor (0/22), as was the submandibular gland. No fusions were detected in *ALK*, *ROS1*, *RET*, *MET*, *N-TRK1*, *N-TRK2*, or *N-TRK3* genes in the Ydilla Genefusion Test. The tumor was composed of epithelioid eosinophilic neoplastic cells with focal cytoplasmic clearing, organized in solid nests, often exhibiting a pseudoalveolar growth pattern (Figure 3A,B) and diffusely expressing S100. The diagnosis MGNET was confirmed. The patient received full-dose postoperative radiotherapy (50 Gy in 25 fractions) with the VMAT technique (Figure 4B,C). During the follow-up period, the patient presented distant metastases in the right gluteal and left paravertebral regions (Figure 5B,C) 3 years after the initial surgery. The patient started systemic chemotherapy with six different lines of treatment due to poor response. The patient died of massive left supraclavicular recurrence with lung involvement (Figure 5A) 54 months after her initial surgery.

## 3. Discussion

Malignant gastro neuroectodermal tumor of the head/neck region is an exceptionally rare neoplasm, with less than 100 cases cited worldwide. Like all rare tumors, diagnosis is significantly challenging due to overlap in histopathological features with other tumors of neuroectodermal origin. The presentation of MGNETs in the head/neck region is often subtle, given that these tumors can grow insidiously without producing specific symptoms until they reach an advanced stage. Patients may present with a mass or swelling, pain, dysphagia, or other region-specific symptoms depending on the exact anatomical location of the tumor. As with all rare tumors, MGNET in this anatomical region further complicates the clinical diagnosis, as it is more commonly associated with the gastrointestinal tract. When histopathology reveals neuroectodermal differentiation but lacks melanocytic markers, MGNET should be included in the differential diagnosis.

The diagnosis of MGNET relies heavily on a combination of histological evaluation and molecular testing. MGNETs display a high degree of cellular atypia and frequent mitotic activity, consistent with their aggressive nature. Necrosis is also common, which contributes to the rapid clinical progression of these tumors. Histologically, MGNET demonstrates a pseudoalveolar or pseudopapillary growth pattern with oval to spindle-shaped cells, eosinophilic cytoplasm, and vesicular nuclei. However, these features overlap with other tumors, such as clear cell sarcoma (CCS), synovial sarcoma, and melanoma, making a definitive diagnosis based on morphology alone difficult. This often delays definitive diagnosis and management, contributing to the poor prognosis associated with these tumors. Radiologically, MGNET may appear as non-specific soft tissue masses, and advanced imaging techniques such as magnetic resonance imaging (MRI) or positron emission tomography (PET) scans are often required to assess the extent of the disease. Immunohistochemistry (IHC) plays a critical role in differentiating MGNET from other entities. MGNET typically shows positivity for S100 and SOX10 while being negative for melanocytic markers like HMB-45, Melan-A, and MiTF, which are usually present in melanoma and CCS. Molecular studies, specifically fluorescence in situ hybridization (FISH), are essential to confirm the presence of EWSR1::CREB1, which are characteristic of MGNET and further distinguish it from other neuroectodermal tumors.

Given its aggressive clinical behavior, early and accurate diagnosis is critical for appropriate treatment planning. Surgical resection remains the cornerstone of treatment for MGNET, aiming for complete excision with negative margins. However, given the anatomical complexity of the head/neck region and the potential for local invasion into critical structures, achieving clear surgical margins can be challenging. Adjuvant therapies, including radiation and chemotherapy, may be considered in cases where complete resection is not possible or in patients with high-risk features such as vascular invasion, perineural spread, or lymph node involvement. Unfortunately, there is no standardized nomenclature or standardized treatment protocols due to the scarcity of cases, and treatment decisions are often based on institutional experience and extrapolation from similar sarcomas. In the future, the fusion itself may name, classify, categorize, and specify treatment.

The prognosis of MGNET in the head/neck is generally poor due to its aggressive nature and high potential for local recurrence and distant metastasis. Factors influencing prognosis include tumor size, mitotic rate, and the ability to achieve complete resection. Long-term survival is uncommon, and patients require close monitoring for recurrence and metastasis. Further research and case studies are needed to improve diagnostic techniques and treatment outcomes for this extremely rare entity.

The management of MGNETs in the head/neck is challenging due to their rarity and aggressive behavior. Surgical resection remains the mainstay of treatment, with the goal of achieving complete excision with clear margins. However, due to the complex anatomy of the head and neck region, radical resection is often difficult without significant functional morbidity. Adjuvant radiotherapy is commonly employed post-surgery to reduce the risk of local recurrence, although its efficacy remains uncertain due to the limited number of cases reported. In the management of MGNET, achieving negative surgical margins remains challenging due to the tumor’s aggressive biological behavior and frequent involvement of adjacent critical anatomical structures. Neither radiation nor chemotherapy has been adequately studied in these extremely rare tumor cases. As intraoperative radiotherapy (IORT) emerges, offering direct delivery of high-dose radiation precisely to the tumor bed, it optimizes local control, particularly in scenarios of microscopic residual disease or in recurrent cases, and may potentially improve long-term oncological outcomes. The role of chemotherapy in MGNET is less defined, largely because these tumors are typically resistant to conventional sarcoma regimens. Nonetheless, some reports suggest that targeted therapies against EWSR1 fusion proteins or other molecular targets may hold promise in the future [19]. Emerging therapies such as immune checkpoint inhibitors or tyrosine kinase inhibitors are currently under investigation in clinical trials, but no definitive treatment guidelines have yet been established [23,24,25].

Furthermore, with this and other rare tumors, precision therapy may be the primary treatment [26]. The prognosis for patients with MGNETs is generally poor due to the tumor’s aggressive behavior and high likelihood of metastasis, particularly to the lungs and regional lymph nodes. Recurrence rates are high, and despite aggressive surgical management, the long-term survival rates remain low. Studies suggest that the 5-year survival rate for patients with MGNETs is less than 30%, emphasizing the need for early detection and multimodal treatment. All information must be shared and disseminated broadly for all Refractory Atypical Resistant Extremely (RARE) tumors. This way similarities and differences may be determined [27,28,29]. Furthermore, for RARE tumors, precision targeted medicine is most important to affect the outcome.

## 4. Conclusions

Extraenteric malignant gastrointestinal neuroectodermal tumor is a rare but highly aggressive malignancy that can present in the head/neck region, posing significant diagnostic and therapeutic challenges. The rarity of this tumor, combined with its overlapping histological features with other neuroectodermal tumors, complicates its diagnosis. Advances in molecular pathology, particularly the identification of EWSR1 translocations, have been crucial in distinguishing MGNET from other malignancies. However, the prognosis remains poor, and further research is needed to better understand the pathogenesis and to develop more effective therapeutic strategies. With the ongoing development of targeted therapies and molecular diagnostics, there is hope that the outcomes for patients with this devastating disease may improve in the future.

## Figures and Tables

**Table 1:** Clinicopathologic and molecular genetic features of published neck E-MGNET cases.

Case	Age/Sex	Location/Size	IHC Results	Molecular Results	Therapy	Local Recurrence	Metastasis	Outcome
1 [18]	9/M	Right neck/5.5 cm	Positive: S100 protein, SOX10, synaptophysin, CD56. Negative: HMB45, Melan A, MART-1, TTF1, EMA, pancytokeratin, cytokeratin AE1/AE3, CAM5.2, CK5/6, p63, CD31, SMA, desmin, synaptophysin, chromogranin, GFAP, CD68	*EWSR1* gene locus was attempted on the specimen but was technically unsatisfactory	Chemotherapy	No	No	ANED at 12 mo
2 [19]	14/M	Right neck/3.7 cm	Positive: S100 protein, SOX10, synaptophysin, CD56; Negative: Keratins, HMB-45, Melan-A, MiTF, chromogranin A, CD117, DOG1, ALK	*EWSR1 exon 8::ATF1 exon 4*	Resection with + margin and radiotherapy	No: persistent local disease, undergoing radiotherapy	No	AWD at 11 mo
3 [19]	30/M	Right neck/NA	Positive: S100 protein, SOX10, synaptophysin, CD56; Negative: Keratins, HMB-45, Melan-A, MiTF, chromogranin A, CD117	*EWSR1 exon 8::ATF1 exon 4*	No radical resection and radiotherapy	Yes: 57 mo	No	ANED at 70 mo
4 [19]	48/F	Right neck/5.5 cm	Positive: S100 protein, SOX10; Negative: Keratins, synaptophysin, MiTF, HMB-45, Melan-A, chromogranin A, CD117, ALK	*EWSR1 exon 7::ATF1 exon 5*	No radical resection	No	No	ANED at 10 mo
5 [our case]	58/F	Left neck/4 cm	Positive: S100, SOX10, CD99 (weak/focal), Fli-1, and synaptophysin (focal). Negative: Melan-A, HMB-45, CKIT, AE1/AE3	*EWSR1* gene translocation	Surgery (radical resection) and radiotherapy	Yes: 48 mo	Yes: 36 mo	Died: 54 mo

Abbreviations: ANED: alive with no evidence of disease; AWD: alive with disease; IHC: immunohistochemistry; E-MGNET: extraenteric malignant gastrointestinal neuroectodermal tumor; mo: months.

## Data Availability

The data presented in this study are available on request from the corresponding author.

## Abbreviations

The following abbreviations are used in this manuscript:

MGNET	malignant gastrointestinal neuroectodermal tumor
CCS	clear cell sarcoma
GI	gastrointestinal
WHO	World Health Organization
IHC	immunohistochemistry
PAS	periodic acid–Schiff (staining)
FISH	fluorescence in situ hybridization
CNIO	Centro Nacional de Investigaciones Oncológicas (National Cancer Research Center, Spain)
PET-CT	positron emission tomography–computed tomography
HPF	high-power field (used in microscopy)
ANED	alive with no evidence of disease
AWD	alive with disease
E-MGNET	extraenteric malignant gastrointestinal neuroectodermal tumor
